# Evidence-based policy responses to strengthen health, community and legislative systems that care for women in Australia with female genital mutilation / cutting

**DOI:** 10.1186/s12978-017-0324-3

**Published:** 2017-05-18

**Authors:** Nesrin Varol, John J. Hall, Kirsten Black, Sabera Turkmani, Angela Dawson

**Affiliations:** 1 Sydney Medical School, Discipline of Obstetrics, Gynaecology and Neonatology, University of Sydney, Sydney, NSW 2006 Australia; 2 School of Public Health and Community Medicine, Faculty of Medicine, University of New South Wales, UNSW Sydney, 2052 NSW Australia; 3 Centre for Midwifery, Child and Family Health, Faculty of Health, University of Technology Sydney, Sydney, NSW Australia

**Keywords:** Female genital mutilation, Cutting, Health policy, Australia, Health systems, Healthcare professional training, Migration, Refugees, Gender-based violence

## Abstract

**Background:**

The physical and psychological impact of female genital mutilation / cutting (FGM/C) can be substantial, long term, and irreversible. Parts of the health sector in Australia have developed guidelines in the management of FGM/C, but large gaps exist in community and professional knowledge of the consequences and treatment of FGM/C. The prevalence of FGM/C amongst Australian women is unknown. Our article reviews the literature on research on FGM/C in Australia, which focuses on health system response to women and girls with FGM/C. Recommendations are made for policy reform in health, legislation, and community programs to provide the best healthcare, protect children, and help communities abandon this harmful practice.

**Main body:**

Midwives and doctors in Australia acknowledged a lack of knowledge on FGM/C, clinical guidelines and consequences for maternity care. In a metropolitan Australian hospital with specialised FGM/C care, women with FGM/C had similar obstetric outcomes as women without FGM/C, underlining the importance of holistic FGM/C clinics.

Greater focus on integration of refugee and migrant populations into their new cultures may be an important way of facilitating the abandonment of this practice, as is education of communities that practise FGM/C, and experts involved in the care and protection of children. Men could be important advocates for protecting women and girls from violence and FGM/C through a man-to-man strategy with programs focussing on men’s health and other personal issues, education, and communication.

The Australian Government has identified gender-based violence as an area of priority and has been implementing a *National plan to reduce violence against women and their children 2010–2022.* A multidisciplinary network of experts on FGM/C could be established within this taskforce to develop well-defined and rapid referral pathways to care for and protect these children, as well as coordinate education and prevention programs to help communities abandon this harmful practice.

**Conclusion:**

Countries of migration can be part of the solution for abandonment of FGM/C through community interventions and implementation of national and coordinated training in FGM/C of experts involved in the care and protection of children and women. The global focus on collaboration on research, training and prevention programs should be fostered between countries of FGM/C prevalence and migration.

## Plain English Summary

With increasing migration in the world, female genital mutilation / cutting (FGM/C) has become a global issue for countries to protect girls from being cut, to provide optimum healthcare for women and girls who have undergone FGM/C, and to help communities abandon this harmful practice. FGM/C can cause serious and long tem complications, which need to be appropriately addressed to prevent further suffering and unnecessary increased cost to the healthcare system. There is very limited data on all aspects of FGM/C in Australia. Two to three percent of women who gave birth at a metropolitan hospital in Australia had FGM/C. Evidence suggests that specialised FGM/C healthcare services are important in providing these women with obstetric and neonatal outcomes that are similar to women without FGM/C [1–4]. Healthcare professionals in Australia had limited knowledge on FGM/C, indicating the need for formal education and training in caring for women with FGM/C. By 2011, it was estimated that there were more than 83,000 women and girls with FGM/C living in Australia [5]. Our article reviews current Australian healthcare and legislative responses in regards to women with FGM/C and suggests evidence-based recommendations for policy reform and prevention programs. Creation of a national, and potentially international, multidisciplinary network of experts involved in the healthcare and protection of children may be an important step forward. Focussing on migrant and refugee integration into their new culture, and involvement of men in prevention programs may also play key roles in helping communities abandon this harmful practice.

## Background

### Why we need a national policy response to FGM/C in Australia

FGM/C is recognised as a form of gender-based violence (GBV) and presents a transnational human rights, gender inequity, and health issue [6, 7]. Worldwide more than 200 million women and girls are living with FGM/C, mainly in 30 African and Middle Eastern countries, as well as in Asia, and in countries of immigration, including Australia and New Zealand [8]. Women and girls with FGM/C can suffer significant and serious long-term physical and psychosexual health problems [9–12]. The prevalence of FGM/C is on the decline globally with a smaller proportion of girls being cut today compared to their mothers’ generation [6]. In countries with data on FGM/C prevalence, 63% of men and 67% of women want the practice to end [8]. However, population growth means that the number of girls and women subjected to FGM/C will increase [13]. A landmark study in 2006 by the World Health Organization found that women with FGM/C have significantly higher maternal and neonatal complications if specialist expertise on problems associated with FGM/C and high quality obstetric services are not available [14]. The substantial costs to the healthcare system associated with managing obstetric complications can be offset by prevention programs and specialist FGM/C health services [15].

FGM/C is a health policy challenge as well as a human rights issue in Australia. In the decade from 1999 to 2009, Australia received about 40,000 people from Sudan, Egypt, Ethiopia and Somalia, countries that have prevalence rates of FGM/C varying from 74 to 98% [6, 16]. By 2011, it was estimated that more than 83,000 women and girls with FGM/C had migrated to Australia from countries in Africa and the Middle East where FGM/C is prevalent [6, 17]. As a result, hospitals in Australia are reporting increased presentations of women with FGM/C for delivery of their babies [18]. Melbourne’s Royal Hospital for Women alone has reported caring for 600 to 700 women with FGM/C annually [19]. Currently there are only three hospitals in Australia that have expertise and policies regarding the care of women with FGM/C. At a metropolitan hospital in Sydney, Australia, providing specialist holistic FGM/C services with gynaecological, obstetric, paediatric, psychological, social work, interpreter, counselling, and de-infibulation services, the prevalence of women with FGM/C who gave birth between 2006 and 2012 was 2 to 3% [20]. In the presence of high quality obstetric care and the specialist FGM/C services provided in this hospital, obstetric and perinatal outcomes for affected women and their babies were similar to women without FGM/C who gave birth at this hospital.

Each state and territory in Australia has enacted specific anti-FGM/C laws since 1994 [21]. It is of great concern that there is evidence that FGM/C is performed in Australia [18], resulting in criminal convictions, with the last case in 2015 [22]. Another case is currently in the courts in Queensland [23]. At the National Summit on FGM/C in 2013, the Australian Government called for improved quality health services for girls and women with FGM/C and stated its commitment to coordinated action for its abandonment through professional and community education campaigns [24]. It recognised the need for better data collection and research on FGM/C in Australia, greater education, especially of men, in communities with FGM/C, along with specific training for health care professionals (HCP), and advocacy for the abandonment of this harmful practice [24]. This review presents evidence-based recommendations that can be used to inform policy and guidelines aimed at strengthening the Australian health, legislative, and community systems to ensure the provision of high quality healthcare, child protection, and FGM/C prevention programs.

### Policy challenges and reform

Policies, guidelines and resources for FGM/C have been developed by most states and territories of Australia and are often similar in their content [25–28]. Although most FGM/C-related health programs are no longer supported by Commonwealth funding, many states and territories continue to provide services to women and communities affected by FGM/C [29]. The reason for the withdrawal of support is unknown but may be related to political priorities. The limited resourcing across Australia has been an important reason that has prevented the development of a comprehensive, national, coordinated, and evidence-based policy response [29]. A national approach could address gaps in Australian research, service provision, and prevention programs. FGM/C must not be accepted as a cultural problem, but rather as a human rights violation, a criminal offence, and a health and policy issue, hence political commitment is required to develop a national approach.

Key components of a national policy should include a response involving the health system, education and child protection sectors, and law enforcement. They would incorporate specialised FGM/C units, clinical practice guidelines for providers, collaboration with and across services, education and training on FGM/C for all healthcare professionals in both urban and rural centres and other professionals involved in the care of women and girls with FGM/C or who are at risk of this procedure. A national data collection system is required to appropriately evaluate services and direct policy response. In addition, strong partnerships with communities should be developed to actively engage members in the design and implementation of advocacy and prevention programs. Finally, national legislation should be developed and enforced, requiring national and international collaboration.

Following the National Summit on FGM/C in 2013, the Australian Government had provided funding for organisations to develop a holistic approach through programs that mobilise and engage communities, and through research and data collection to build the evidence to support women and girls affected by FGM/C in Australia. It underlined the importance of community empowerment, women’s leadership, the role of men, health system strengthening, and a review of the legal framework in Australia.

### The Australian health system response

Currently there are only three hospitals in Australia that have specialised FGM/C clinics with clinical practice policies, namely Auburn Hospital in Sydney, the Royal Hospital for Women in Melbourne and King Edward Memorial Hospital in Perth [30–32]. There are other hospitals in Victoria where the Family and Reproductive Rights Program workers provide consultation, education and training for service providers, offer referrals and support for women affected by FGM/C and undertake specific projects in partnerships with relevant organisations [33]. The NSW Ministry of Health has developed guidelines for maternity care of women with FGM/C, which can be adopted nationally. Clinical practice guidelines form the framework for best medical and nursing practice, and underpin training and education requirements for HCP. We need to establish further specialised FGM/C clinics in hospitals and health care services where women with FGM/C present. These clinics should be holistic and incorporate existing gynaecological, urological, sexual dysfunction, psychological, paediatric, interpreter, and social work services in urban hospitals in Australia. This would alleviate the burden of disease among affected women and reduce healthcare costs for the Government [15]. Family Planning Victoria has outlined the need to develop partnerships between healthcare facilities that provide care to women with FGM/C and other community and government services [28]. Local, regional, and national collaborations are required for rapid referrals to experts who provide the best holistic care for these women and girls, and leverage community prevention to protect the children [28].

Policy development and appropriate allocation of resources and services require data on prevalence and burden of disease. This information also underpins monitoring and evaluation of interventions. The demographic and health surveys undertaken in low- and middle-income countries gather information on prevalence of FGM/C and associated health problems. In Australia, however, the census does not collect health statistics, and to date the only prevalence data we have is from our study of women with FGM/C who gave birth at a metropolitan hospital [20]. Hospitals and healthcare facilities in Australia are probably the most practical and accessible way to obtain nationwide standardised data on the prevalence of women with FGM/C, albeit women who present for medical care, the actual risk of being cut to girls born to mothers with FGM/C, and the burden of complications from FGM/C. We need to include information on this practice in national maternal and perinatal morbidity data collections as part of the National Perinatal Data Collection of the Australian Institute of Health and Welfare. This would assess the presence of complications and obstetric and neonatal outcomes.

Education and training on the sociocultural underpinnings of FGM/C, management of its complications, and obstetric management, are a prerequisite for accurate and consistent data collection, as well as for the provision of specialised healthcare services [18, 34, 35]. Midwives in Australia were cognisant of their lack of confidence and fear of caring for women with FGM/C due to cultural misunderstanding and difficulty in development of rapport, lack of knowledge about FGM/C and data collection, and ignorance of relevant clinical guidelines and policies [36]. These findings were supported by the experiences of African refugee women with FGM/C giving birth in Australia [37]. They were concerned about the cultural competence, experience and training of Australian midwives and obstetricians, and hence felt the need to explain the management of FGM/C during labour in their countries of origin.

A survey of about 500 child health specialists in Australia showed that 10% had seen at least one girl under the age of 18 with FGM/C during their career [38]. Only 15% of these clinicians acknowledged they had some education and training in FGM/C and 65% requested educational materials on FGM/C [38]. Global experience on education and training for HCP involved in the care of women with FGM/C is in keeping with findings in Australia [34, 35, 39–42]. In response to this lack of knowledge, training courses and education modules on FGM/C for continuous professional development for HCPs have been developed by various organisations in different states in Australia [25, 26, 31, 43, 44]. These should be formally incorporated into the curricula of under- and postgraduate medical, midwifery and nursing colleges.

### Change from within communities

Multi-pronged programs with community-led initiatives in conjunction with a legal response have been found to have success in addressing FGM/C. The largest decline in practice globally has been seen in Kenya and Burkina Faso where there has been a very strong legal as well as community education response [6, 45–47].

A systematic review of the evaluation of effectiveness of eight controlled intervention studies in Africa to prevent FGM/C showed that education through information dissemination, including the health complications of FGM/C and reproductive function, is probably advantageous for successful change for communities to abandon this harmful practice by questioning its validity [48]. Whilst the meta-analyses results revealed an uncertainty between these variables, study-level results showed a positive relationship between the empowerment and community interventions and knowledge on health consequences. Moreover, they affected participants’ beliefs about the benefits of the practice, their approval, regrets about having their daughters cut, and intentions to subject their daughters to FGM/C. The limited effectiveness of the studies were considered to be likely due to imperfect relevance and implementation fidelity [48]. The former refers to the ill fit between an intervention and the sociocultural and demographic characteristics of the target community [49]. Programs need to be community-led using local resources and key opinion leaders, and tailored to take into consideration the ideological structure, ethnic and socioeconomic differences of each community [9, 48, 50]. Responding to communities’ needs and priorities would play an integral part in gaining trust and making change relevant for them [51].

Studies have shown that migration of people to countries where FGM/C is not prevalent has a positive influence on the abandonment of this practice [52–56]. Reasons are likely to be the weakening of social pressure and removal of benefits of the practice in regards to marriageability and social acceptance. FGM/C is a prosecutable offence and becomes a disadvantage or stigma for girls in non FGM/C-practising countries. Australia and other countries of migration can hence play an important role in fostering the change to abandonment nationally and internationally through community-led education programs. Programs should be holistic and incorporate resettlement issues, and education on gender relations, domestic violence, reproductive health, and human rights [57]. Programs must be supported by child protection and law enforcement measures to protect girls because support for FGM/C can also be highly intractable. There is evidence that FGM/C is sometimes adopted by new groups and in new areas after migration [58].

#### Migration and resettlement

FGM/C and its associated complications are usually not the primary problems for women who arrive to Australia as refugees or migrants. Women and girls who are refugees are very likely to have come from conflict zones where they experienced poverty, malnutrition, health problems, lack of educational opportunities and limited access to health services [59]. On resettlement in Australia, refugees as well as other migrant women face major challenges because of poor or no English skills, low education levels and limited financial resources, which inhibit access to government services, integration and assimilation [60]. Moreover, they may experience loneliness and isolation due to lack of social support, difficulty accessing affordable accommodation, or domestic violence. Gender roles may change and women may acquire a new status in society [61, 62]. Their partners may find these changes unacceptable as they threaten the patriarchy. They may respond with violence and family breakdown may occur. A community-based participatory study that examined the reproductive health priorities of 319 refugee women in Australia and New Zealand found that these women were mainly concerned about their economic problems such as maintaining basic resources for resettlement, the racial and social problems of their children, intergenerational family conflict, and their remaining family in conflict areas [63].

Meaningful and successful engagement with women who experienced FGM/C and with their families requires an understanding of the socio-cultural imperatives for FGM/C. Refugee women in Australia and New Zealand felt resentful about being identified and perceived solely as “infibulated women” as a result of the national and international profile of FGM/C abandonment programs [63]. These women were subjected to FGM/C as children when they were unable to give consent. Parents cut their daughters for social acceptance, marriageability, and fear of exclusion from resources and opportunities as a young woman [50]. The inference for Australia and other countries of migration is for government policies to support services to care for these women and girls and their families, as well as invest in programs that allow better integration of refugees and migrants into society and participation in community life.

#### The role of men

FGM/C affects men as well as women [64]. A systematic review on the role of men in FGM/C suggests that many men felt they, too, were victims of this practice and wanted to see it end [65]. Social obligation was reported as an important barrier to stopping FGM/C. Higher level of education was one of the most important indicators for men’s support for abandonment of FGM/C [65]. In Guinea, Sierra Leone and Chad, for example, more men than women wanted FGM/C to end [6].

Studies show that men generally want and respond positively to be involved in sexual and reproductive health programs [66, 67]. However, men’s involvement in reproductive health services generally has been primarily for the benefit of women [67, 68]. In Australia, a more positive and successful involvement of men in the abandonment of FGM/C and GBV may be achieved by the provision of reproductive health services specific for men. Men-only programs with a man-to-man strategy could be explored with a focus on male reproductive and general health, other private issues, and health literacy to empower men to make informed and healthy decisions for themselves and their families [65]. The involvement of men should complement current programs focusing on education and empowerment of girls and women. Programs for men and women need to work together to address reproductive health issues, FGM/C, GBV, parenting strategies, as well as communication and relationship skills. Influential people in the community could lead programs as advocates and facilitate dialogue between men and women, their communities, and government bodies [65].

### Legislation

FGM/C is banned by law in 23 African countries [69]. FGM/C was first made illegal in New South Wales (NSW) in 1994, and subsequently every state and territory has enacted specific anti-FGM/C laws. Penalties at present vary greatly, ranging from 7 years imprisonment in some jurisdictions up to 21 years’ imprisonment in others [21]. Implementation of the law can itself be a highly effective form of education, as the recent successful prosecution of three people in NSW has shown. It had global implications for the members of the Dawodi Bohra Muslim community. It allowed its members to speak out about this practice and spurred an international campaign to end the practice [70].

Legislation on its own will not stop FGM/C, and the practice will continue underground as long as there is demand. Legislation and prosecution are best placed within an integrated, holistic framework of culturally affirming interventions based on human rights [57]. Similar to the European Union, in Australia the broad network of professionals required for prosecuting cases has limited tools for education and training on FGM/C [7]. We need multi-disciplinary national programs that are integrated into the training of professionals involved in child protection on how to identify a girl at risk, mandatory reporting laws for children at risk or those who have been cut, and appropriate and rapid referral pathways to the appropriate agencies for investigation and protection. The details of the required child protection measures are beyond the scope of this paper.

### National and international collaborations

The transnational presence of FGM/C calls for an international collaboration on research, training and prevention programs. The University of Sydney has been involved in establishing an Africa Coordinating Centre for abandonment of FGM (ACCAF) at the University of Nairobi, Kenya, in 2012 [71]. Its mandate is to contribute to the abandonment of FGM within Africa and beyond through coordination of multinational and trans-disciplinary innovative research, training/capacity building, dissemination of evidence based practices and strategies for abandonment of FGM/C, influencing policy, and behaviour change [71]. The Australian and other Government(s) and non-government organisations globally can collaborate with ACCAF to share expertise and resources.

In 2013, the National Summit on FGM/C at Parliament House in Canberra had already brought together many of the experts involved in the healthcare and protection of women and girls, and prevention programs to establish an agreed way forward for coordinated action [24]. It is important that this coalition of professionals advocate for the development of a national policy. Similar responses through multi-agency cooperation have been instituted in the EU Member States [7] such as the UK ‘FGM Multi-Agency Practice Guidelines’ [72] and the ‘Chain Approach’ (Ketenaanpak) in the Netherlands [73]. Similar to our need in Australia, the European Institute for Gender Equality Report on FGM/C calls for continual, structured and nationwide training on FGM/C, as well as data collection [7].

The Australian Government has addressed violence against women as one of their areas of focus and has been implementing a 12-year *National Plan to reduce violence against women and their children 2010 – 2022* [74]. The National Plan focuses on domestic and family violence and sexual assault with prevention and intervention programs involving women, men and communities, provision of support services for women who have experienced violence, and research and evaluation of programs to inform policy. The agenda is holistic and incorporates other national reforms on children, and settlement services for refugee and migrant women, as well as trafficking and human slavery, disability, and homelessness. Australia has strong and world-class legal, human rights, health and education systems. There is the potential for HCPs, teachers, welfare officers, child protection officers, government and non-government organisations involved in prevention programs on FGM/C, the police, and the judicial sector in Australia to form a network of experts within this National Plan to coordinate research, training and prevention programs, as well as policy reform.

## Conclusion

FGM/C, within the broader context of GBV, is a transnational human rights and health issue. An understanding of the complex socio-cultural imperatives of this practice is important to guide government policy and guidelines to best care for and protect women and girls from this practice. There needs to be political commitment to support changes to the health system, and training of professionals involved in the protection and care of children and women.

Australia can play a leading role in the protection of children from this harmful practice. Migration and education are potent catalysts for abandonment. This can be facilitated by policy that prioritises migrants’ and refugees’ social and economic integration and participation in community life, increasing access to health and education, and addressing discrimination and inequity. A multidisciplinary network of experts on FGM/C within the Australian *National Plan to reduce violence against women and children 2012–2022* could establish defined and rapid referral pathways to protect girls and coordinate education and prevention programs to help communities abandon this harmful practice.

## Abbreviations

ACCAF: Africa Coordinating Centre for abandonment of female genital mutilation
FGM/C: Female genital mutilation / cutting
GBV: Gender-based violence
HCP: Healthcare professionals

## References

[1] Amusan OA, Asekun-Olarinmoye EO (2006). Knowledge, beliefs, and attitudes to female genital mutilation (FGM) in Shao Community of Kwara State, Nigeria. Int Q Community Health Educ.

[2] Essen B, Sjoberg N-O, Gudmundsson S, Ostergren P-O, Lindqvist P (2004). No association between female circumcision and prolonged labour: a case control study of immigrant women giving birth in Sweden. Eur J Obstet Gynecol Reprod Biol.

[3] Wuest S, Raio L, Wyssmueller D, Mueller M, Stadlmayr W, Surbek DV (2009). Effects of female genital mutilation on birth outcomes in Switzerland. BJOG.

[4] Abdelshahid A, Campbell C (2015). Should I circumcise my daughter?" Exploring diversity and ambivalence in Egyptian parents’ social representations of female circumcision. J Community Appl Soc Psychol.

[5] No FGM Australia: New report: 3 girls a day at risk of FGM. 25 March 2014. Available at: http://www.nofgmoz.com/2014/03/25/new-statistics-of-girls-at-risk-of-fgm-in-australia. Accessed 23 Mar 2017.

[6] United Nations Children’s Fund. Female genital mutilation/cutting: A statistical overview and exploration of the dynamics of change. UNICEF, New York. July 2013. Available at: http://www.unicef.org/publications/index_69875.html. Accessed 16 June 2015.

[7] van Vossole A, Middelburg MJ, Arnaut C, Leye E, Deblonde J, Mergaert L, et al. Female genital mutilation in the European Union and Croatia. European Institute for Gender Equality. Report. 2013. Available at: http://eige.europa.eu/rdc/eige-publications/female-genital-mutilation-european-union-report. Accessed 26 July 2016.

[8] United Nations Children's Fund. Female genital mutilation/cutting: a global concern. UNICEF, New York, 2016. Available at: https://www.unicef.org/media/files/FGMC_2016_brochure_final_UNICEF_SPREAD.pdf. Accessed 29 July 2016.

[9] World Health Organization. Eliminating female genital mutilation: An interagency statement–OHCHR, UNAIDS, UNDP, UNECA, UNESCO, UNFPA, UNHCR, UNICEF, UNIFEM, WHO. 2008. Available at: http://www.who.int/reproductivehealth/publications/fgm/9789241596442/en/. Accessed 16 June 2015.

[10] Almroth L, Bedri H, El Elmusharaf S, Satti A, Idris T, Hashim MSK, et al. Urogenital complications among girls with genital mutilation: A hospital based study in Khartoum. Afr J Reprod Health 2005a, 9:127–133. 16485592

[11] Talle A, Hernlund Y, Shell-Duncan B (2007). Female circumcision in Africa and beyond: the anthropology of a difficult issue. Transcultural bodies: female genital cutting in global context.

[12] Elnashar RA, Abdelhady R (2007). The impact of female genital cutting on health of newly married women. Int J Gynaecol Obstet.

[13] United Nations Children’s Fund. Female genital mutilation/cutting: What might the future hold? 2014. Available at: http://reliefweb.int/sites/reliefweb.int/files/resources/FGM-C_Report_7_15_Final_LR.pdf. Accessed 16 June 2015.

[14] Banks E (2006). Female genital mutilation and obstetric outcome: WHO collaborative prospective study in six African countries. Lancet.

[15] Adam T, Bathija H, Bishai D, Bonnenfant YT, Darwish M, Huntington D (2010). Estimating the obstetric costs of female genital mutilation in six African countries. Bull World Health Organ.

[16] Australian Government Department of Immigration and Citizenship (2009). Settler arrivals 1998-99 to 2008-09, Australia, states and territories.

[17] Australian Bureau of Statistics: Migration, Australia, 2010-11. In*.* Canberra / ABS. Cat. No. 3412.0; 2010.

[18] Moeed S, Grover S (2012). Female genital mutilation/cutting (FGM/C): Survey of RANZCOG Fellows, Diplomates & Trainees and FGM/C prevention and education program workers in Australia and New Zealand. ANZJOG.

[19] Bourke E. Female circumcision happening in Australia. ABC News. 2010.Updated 6 Feb 2010. Available at: http://www.abc.net.au/news/stories/2010/02/06/2812147.htm. Accessed 8 June 2015.

[20] Varol N, Dawson A, Turkmani S, Nanayakkara S, Hall J, Homer CSE (2016). Obstetric outcomes for women with female genital mutilation at an Australian hospital, 2006-2012 - A descriptive study. BMC Pregnancy Childbirth.

[21] Review of Australia's female genital mutilation framework. Final Report. Australian Government, Attorney General's Department. Available at: https://www.ag.gov.au/Publications/Pages/ReviewofAustraliasFemaleGenitalMutilationlegalframework-FinalReportPublicationandforms.aspx. Accessed 23 Mar 2017.

[22] Gardiner S. Mother, midwife and sheikh guilty in Australia's first genital mutilation trial. Sydney Morning Herald. 2015 Nov 12. Available at: https://www.smh.com.au/nsw/mother-midwife-and-sheikh-guilty-in-australias-first-genital-mutilation-trial-20151112-gkx0b3.html. Accessed 23 Mar 2017.

[23] Brisbane couple to stand trial over girls’ genital mutilation. Brisbane Times. 13 Apr 2017. Available at: http://www.brisbanetimes.com.au/queensland-couple-to-stand-trial-over-girls-genital-mutilation-20160413-go5s4j.html. Accessed 23 Mar 2017.

[24] National Summit on female genital mutilation. Minister Plibersek's speech at the National Summit on Female Genital Mutilation. Available at: https://women.wcha.asn.au/news/national-summit-female-genital-mutilation-speech. Accessed 9 Apr 2013.

[25] Female Genital Mutilation (FGM) in clinical practice DVD. A resource for health professionals. Family Planning Queensland. 2008. Available at: http://www.true.org.au/Resources/shop#!/Female-genital-mutilation-FGM-in-clinical-practice-DVD/p/62799432. Accessed 15 Oct 2015.

[26] Royal Australian and New Zealand College of Obstetricians and Gynaecologists. Female genital mutilation education resource. CLIMATE e-learning. August 2015. Available at: https://www.climate.edu.au. Accessed 15 Sept 2015.

[27] Maternity-Pregnancy and Birthing Care for Women Affected by Female Genital Mutilation / Cutting. NSW Kids and Families. NSW Government, Ministry of Health. 23 September 2014. Available at: http://www1.health.nsw.gov.au/pds/ActivePDSDocuments/GL2014_016.pdf. Accessed 2 June 2015.

[28] Lynne J, Neopytou K, James C. Family Planning Victoria. Improving the health care of women and girls affected by female genital mutilation/cutting. A national approach to service coordination. 2014. Available at: http://www.fpv.org.au/assets/resources/FGM-ServeCoOrdinationGuideNationalWeb.pdf. Accessed 30 July 2016.

[29] Chen J, Quizon R. NETFA Best practice guide for working with communities affected by FGM/C. Multicultural Centre for Women's Health, Melbourne. 2014. Available at: http://www.netfa.com.au/national-education-toolkit-for-fgm-c-awareness-best-practice-guide.php. Accessed 23 Mar 2017

[30] Auburn Hospital. Western Sydney Local Health District. NSW Government Health. Available at: http://www.wslhd.health.nsw.gov.au/Auburn-Hospital. Accessed 17 Nov 2015.

[31] Female Genital Mutilation FGM - Issues for Clinical Practice. The Royal Children's Hospital, Melbourne. Available at: http://www.rch.org.au/erc/media_production/Female_Genital_Mutilation_FGM_-_Issues_for_Clinical_Practice/. Accessed 23 Mar 2017.

[32] Female genital mutilation - Women and Newborn Health Service, King Edward Memorial Hospital. Clinical Guidelines - Obstetrics & Midwifery. Sept 2014. Available at: http://www.kemh.health.wa.gov.au/development/manuals/O&G_guidelines/sectionb/1/b1.3.pdf. Accessed 15 Nov 2015.

[33] Family and Reproductive Rights Education Program (FARREP). Multicultural Centre for Women's Health. Available at: http://www.mcwh.com.au/FARREP.php. Accessed 23 Mar 2017.

[34] Dawson A, Homer CSE, Turkmani S, Black K, Varol N (2015). A systematic review of doctors’ experiences and needs to support the care of women with female genital mutilation. Int J Gynecol Obstet.

[35] Dawson A, Turkmani S, Fray S, Nanayakkara S, Varol N, Homer C (2015). Evidence to inform education, training and supportive work environments for midwives involved in the care of women with female genital mutilation: A review of global experience. Midwifery.

[36] Dawson A, Turkmani S, Varol N, Nanayakkara S, Sullivan E, Homer CS (2015). Midwives’ experiences of caring for women with female genital mutilation: Insights and ways forward for practice in Australia. Women Birth.

[37] Murray L, Windsor C, Parker E, Tewfik O (2010). The experiences of African women giving birth in Brisbane, Australia. Health Care Women In.

[38] Sureshkumar P, Zurynski Y, Moloney S, Raman S, Varol N, Elliott E (2016). Female genital mutilation in children: Survey of paediatricians’ knowledge, attitudes and practice. Child Abuse Neglect.

[39] Zaidi N, Khalil A, Roberts C, Browne M (2007). Knowledge of female genital mutilation among healthcare professionals. Journal of Obstetrics & Gynecology.

[40] Widmark C, Levál A, Tishelman C, Ahlberg BM (2010). Obstetric care at the intersection of science and culture: Swedish doctors’ perspectives on obstetric care of women who have undergone female genital cutting. Journal of Obstetrics & Gynaecology.

[41] Tamaddon L, Johnsdotter S, Liljestrand J, Essen B (2006). Swedish health care providers’ experience and knowledge of female genital cutting. Health Care for Women International.

[42] Lazar J, Johnson-Akgakwu C, Davis O, Shipp M. Providers’ perceptions of challenges in obstretrical care for Somali women. Obstetrics and Gynecology International, vol. 2013, Article ID 149640, 12 pages, 2013. doi:10.1155/2013/149640 10.1155/2013/149640 PMC3816065 24223041

[43] NSW Education Program on female genital mutilation. Updated 6 Nov 2014. Available at: http://www.dhi.health.nsw.gov.au/NSW-Education-Program-on-Female-Genital-Mutilation/NSW-Education-Program-on-Female-Genital-Mutilation/default.aspx. Accessed 18 June 2015.

[44] Family Planning Alliance Australia (FPAA) National Certificate in Reproductive & Sexual Health for doctors. 2015. Available at: https://www.fpnsw.org.au/education-training/courses/fpaa-national-certificate-reproductive-sexual-health-doctors-distance. Accessed 26 2017.

[45] National Council for Law Reporting, Prohibition of Female Genital Mutilation Act. Available at: http://www.kenyalaw.org/kl/fileadmin/pdfdownloads/Acts/ProhibitionofFemaleGenitalMutilationAct_No32of2011.pdf. Accessed 23 Mar 2017.

[46] Shell-Duncan B, Hernlund Y, Moreau A (2013). Legislating Change? Responses to criminalizing female genital cutting in Senegal. Law Society Review.

[47] Rahman A, Toubia N (2000). Female Genital Mutilation: A practical guide to worldwide laws and policies.

[48] Berg E, Denison E (2012). Effectiveness of interventions designed to prevent female genital mutilation/cutting: a systematic review. Stud Family Plann.

[49] McKenzie JF, Smeltzer JL (2001). Planning, Implementing and Evaluating Health Promotion Programs.

[50] United Nations Children's Fund (UNICEF). Changing a harmful social convention: female genital mutilation/cutting. In*.* Innocenti Digest No. 12. 2005. Available at: https://www.unicef-irc.org/publications/396/. Accessed 26 Mar 2017.

[51] Varol N, Fraser IH, Ng C, Jaldesa G, Hall J (2014). Female genital mutilation/cutting: towards abandonment of a harmful traditional practice. Aust NZ J Obstet Gynaecol.

[52] Gele A, Kumar B, Hjelde K, Sundby J (2012). Attitudes towards female circumcision among Somali immigrants in Oslo: a qualitative study. Int J Womens Health.

[53] Gele A, Johansen E, Sundby J (2012). When female circumcision comes to the West: Attittudes toward the practice among Somali Immigrants in Oslo. BMC Public Health.

[54] Johnson-Agbakwu CE, Helm T, Killawi A, Aasim I (2014). Perceptions of obstetrical interventions and female genital cutting: insights of men in a Somali refugee community. Ethn Health.

[55] Johnsdotter S, Moussa K, Carlbom A, Aregai R, Essén B (2009). “Never my daughters”: A qualitative study regarding attitude change toward female genital cutting among Ethiopian and Eritrean families in Sweden. Health Care Women Int.

[56] Mitike G, Deressa W (2009). Prevalence and associated factors of female genital mutilation among Somali refugees in eastern Ethiopia: a cross-sectional study. BMC Public Health.

[57] Costello S, Quinn M, Tatchell A, Jordan L, Neophytou K. A Tradition in Transition: Female genital mutilation/cutting. A literature review, an overview of prevention programs and demographic data for Victoria, Australia, Mar 2013. Family Planning Victoria. Available at: http://www.fpv.org.au/assets/resources/PreventionProgramsFINALRMITreportMay-10Web1.pdf.

[58] Abusharaf R, ed. Female circumcision: multicultural perspectives. 2007. Philadelphia, University of Pennsylvania Press. 2007.

[59] Peril or protection: the link between livelihoods and gender based violence in displacement settings. Women's Refugee Commission. 1 November 2009. Available at: https://womensrefugeecommission.org/resources/document/564-peril-or-protection-the-link-between-livelihoods-and-gender-based-violence-in-displacement-settings. Accessed 31 May 2015.

[60] Dona G, Berry JW, Ager A (1999). Refugee acculturation and reacculturation. Refugees: Perspectives on the experience of forced migration.

[61] Fact Sheet 5: Refugee Women. NSW Refugee Health Service. NSW Health. March 2011. Available at: https://www.swslhd.nsw.gov.au/refugee/pdf/Resource/FactSheet/FactSheet_05.pdf. Accessed 30 May 2015.

[62] Comas-Diaz L, Jansen MA (1995). Global conflict and violence against women. Peace Conflict J Peace Psychol.

[63] Guerin PB, Allotey P, Elmi FH, Baho S (2006). Advocacy as a means to an end: assisting refugee women to take control of their reproductive health needs. Women Health.

[64] Almroth L, Almroth-Berggren V, Hassanein OM, Al-Said SSE, Hasan SSA, Lithell U-B (2001). Male complications of female genital mutilation. Soc Sci Med.

[65] Varol N, Turkmani S, Black K, Hall J, Dawson A (2015). The role of men in abandonment of female genital mutilation: a systematic review. BMC Public Health.

[66] Baylies C, Bujra J (2000). AIDS, Sexuality and Gender in Africa; the Struggle Continues; Collective Strategies for Protection Against AIDS in Tanzania and Zambia.

[67] Drennon M (1998). Reproductive Health. New Perspectives on Men's Participation. Popul Rep J Family Plan Progams.

[68] Sternberg P, Hubley J (2004). Evaluating men's involvement as a strategy in sexual and reproductive health promotion. Health Promot Int.

[69] United Nations Population Fund. Female genital mutilation (FGM) frequently asked questions. 2015. Available at: http://www.unfpa.org/resources/female-genital-mutilation-fgm-frequently-asked-questions - banned_by_law. Accessed 26 Mar 2017.

[70] Tavawalla D. The practice of khatna or female genital mutilation amongst the Dawoodi (Daudi) Bohra Shia Muslim community - Part 1. 2016 Jan 17. Available at: https://dat2016blog.wordpress.com/2016/01/17/the-practice-of-khatna-or-female-genital-mutilation-amongst-the-dawoodi-bohra-shia-muslim-community-part-1/. Accessed 24 Mar 2017.

[71] Africa Coordinating Centre for Abandonment of FGM/C. 2014. Available at: http://accaf.uonbi.ac.ke/. Accessed 8 June 2015.

[72] Multi-Agency Practice Guidelines: Female Genital Mutilation. HM Government. 2014. Available at: https://www.gov.uk/government/uploads/system/uploads/attachment_data/file/380125/MultiAgencyPracticeGuidelinesNov14.pdf. Accessed 21 June 2015.

[73] European Institute for Gender Equality. Good practices in combating female genital mutilation. 6 Mar 2013. Available at: http://eige.europa.eu/rdc/eige-publications/good-practices-combating-female-genital-mutilation. Accessed 26 Mar 2017.

[74] The National Plan to reduce violence against women and their children 2010-2022. An Initiative of the Council of Australian Governments. 2011. Available at: https://www.dss.gov.au/our-responsibilities/women/programs-services/reducing-violence/the-national-plan-to-reduce-violence-against-women-and-their-children-2010-2022. Accessed 18 Oct 2015.

